# Psychometric properties of the Portuguese version of the physical activity parenting practices questionnaire

**DOI:** 10.1186/s40359-023-01444-4

**Published:** 2023-11-28

**Authors:** Marco Silva-Martins, Ana Catarina Canário, Isabel Abreu-Lima, Lindon Krasniqi, Orlanda Cruz

**Affiliations:** 1 Faculty of Psychology and Education Sciences, University of Porto, Rua Alfredo Allen, 4200-135 Porto, Portugal; 2 University of Trento, via Calepina, 14-38122 Trento, Italy

**Keywords:** Physical activity encouragement, Physical activity discouragement, Physical activity parenting practices, Childhood overweight and obesity, Confirmatory factor analysis, Measurement invariance

## Abstract

**Background:**

Adopting a healthy lifestyle, including regular physical activity, is often part of interventions targeting childhood overweight and obesity. However, to properly inform the objectives of the intervention, reliable psychometric measures are needed to better understand children’s and their families necessities and characteristics.

**Objectives:**

To evaluate the psychometric properties of the Physical Activity Parenting Practices questionnaire in a community sample of Portuguese parents of children aged 5–10, assess measurement invariance across children’s weight status, and construct validity.

**Methods:**

Five hundred three parents completed the Portuguese version of the Physical Activity Parenting Practices (PAPP) questionnaire, a sociodemographic questionnaire, the Comprehensive Feeding Practices Questionnaire, and the Lifestyle Behavior Checklist. A subsample (*n* = 125) completed the PAPP questionnaire 1 month later. Data analyses were performed using R’s lavaan (version 0.6–12) and psych (version 2.2.9) packages.

**Results:**

Confirmatory factor analyses revealed good psychometric properties for the PAPP’s single-factor Encouragement scale and the three-factor Discouragement scale. Configural, metric, and scalar invariance were found across parents of children with different weight statuses for both scales’ factor structures. Internal reliability ranged from α = .64 to α = .89, and test-retest reliability ranged from *r* = .57 to *r* = .74.

**Conclusions:**

The constructs evaluated by PAPP questionnaire revealed adequate validity. The Portuguese version of the PAPP questionnaire is a reliable measure to assess relevant physical activity parenting practices, capable of differentiating the practices of parents with children of different weight statuses, and useful for both research and intervention purposes.

**Supplementary Information:**

The online version contains supplementary material available at 10.1186/s40359-023-01444-4.

## Background

Childhood overweight (OW) and obesity (OB) are major public health challenges with an increasing prevalence worldwide over the last decades [1]. The negative impact of OW/OB on quality of life since early ages is well documented, as well as its association with other physical and psychological negative health conditions throughout the life course [2].

OW/OB often results from an imbalance between energy consumption and expenditure and therefore can be prevented by adopting a healthy lifestyle, including regular physical activity (PA) [3]. Regular PA throughout childhood has been associated with many benefits to children’s health, development, and well-being [4]. Also, there is evidence that early engagement in PA tends to persist over life, with higher levels of PA in childhood predicting higher levels of PA across adolescence and adulthood [5]. Thus, it is crucial to understand how to promote PA in children, fostering efficacy in preventing OW/OB throughout the lifespan.

Childhood OW/OB and related diseases are largely preventable [2]. Parents play a central role in children’s health behaviors, namely by encouraging or discouraging PA through their parenting practices [6, 7]. Several studies suggest that PA-related parenting practices may be associated with children’s weight status [8–10]. A systematic review by Loprinzi et al. [10] points out that parents of children with higher weight status are more likely to provide less support for children’s PA. Yet, the authors acknowledge that it remained unclear whether these parents did use ineffective parenting practices more often, or whether children with these characteristics were less likely to respond favorably to such practices [9, 10]. The literature is not clear on the relation between PA parenting practices and children’s body mass index (BMI) or weight status. Some studies found positive [11], or no associations [8] between parental support and young children’s weight, whereas a longitudinal study found a positive association between parental positive control, general parental encouragement, and overall parental support for PA at baseline and children’s PA 7 months after, for children with low and medium BMI z-scores [9].

The way specific parenting practices affect children’s engagement in PA requires further investigation and, in this regard, reliable and valid measures are crucial [12, 13]. Valid measures in this domain are also relevant to assess the results of parenting interventions focused on parents as the main agents of change on children’s PA [12, 13].

The expert-informed PA parenting practices conceptual framework is empirically validated and developed to build a repository of relevant items to assess PA parenting practices [12]. The categorization of key PA parenting practices was informed by a taxonomy developed by previous research in the field of PA [14]. The validation of the initial item bank provided nine constructs, distributed across three domains: neglect/control, autonomy promotion, and structure [12]. The neglect/control domain comprises one single construct, coercive control, that refers to ineffective parenting practices organized in a continuum [12]. One end of this continuum includes practices characterized by low demands over children’s PA-related behaviors, reflecting a lack of rules and structure, whereas the other end refers to practices reflecting forceful demands (e.g., pressuring, shaming, threatening, punishing), that contribute to undermine children’s intrinsic motivation [15]. The autonomy promotion domain includes parenting practices reflecting autonomy support, guided choice and rewards for children’s engagement in PA [12]. Finally, the structure domain refers to parenting practices reflecting non-directive support and logistic organization of children’s environment to promote children’s involvement in PA [12].

A conceptual framework was also developed in the context of child feeding practices [16]. For this purpose, a group of experts was consulted to develop a content map, considering 28 feeding parenting practices based on existing measures [16], such as the Comprehensive Feeding Practices Questionnaire [17] used in the current study. The final taxonomy included three domains: coercive control (e.g., restriction, pressure to eat, food to control emotions, food-based threats and bribes), structure (e.g., rules and limits, guided choices, monitoring, modeling, health environment), and autonomy support (e.g., nutrition education, encouragement, involvement). Efforts to use consistent terminology in the conceptualization of PA parenting practices and feeding parenting practices were highly recommended by previous research, and can contribute to align the research on feeding and PA parenting practices within a consistent framework [7].

The Preschooler Physical Activity Parenting Practices (PAPP) is a self-report measure to identify practices used by parents to encourage and/or discourage pre-school children’s engagement in PA [7, 18]. In its first study the measure was used in a Latino sample of parents of pre-school children [7]. Other studies also used the measure with parents or caregivers of pre-school children [19, 20], but not of older children, such as school-aged children.

In its original version [7], the measure presents two independent scales. The Encouragement scale (PAPP-E) is comprised of the single-factor Engagement and Structure, and reflects parenting practices characterized by involvement and responsiveness to children’s PA. The Engagement and Structure factor includes items from the PA parenting practices’ conceptual framework [12], namely the Autonomy Promotion and the Structure domains. The Discouragement scale (PAPP-D) reflects parenting practices that sustain children’s sedentary behavior and discourage children’s engagement in PA, and includes four factors: Promote Screen Time, Promote Inactivity, Psychological Control, and Restriction for Safety Concerns [7]. The factors Promote Screen Time and Promote Inactivity refer to practices characterized by high parental permissiveness regarding screen time and PA, and lack of guidance, corresponding to the lower end of the control continuum of the neglect/control dimension of the PA parenting practices’ conceptual framework [12]. The Psychological Control factor stands for parents’ use of pressuring, shaming, and threatening to reduce children’s PA and corresponds to the higher end of the neglect/control dimension from the PA parenting practices’ conceptual framework [12], whereas the Restriction for Safety Concerns factor regards parental restriction of children’s PA due to concerns with their safety [7]. This is consistent with the Structure domain of the above-stated framework.

A key concept related to parenting practices adopted by parents to manage children’s PA and feeding-related behaviors is parental self-efficacy. The use of ineffective parenting strategies is associated with low levels of parental self-efficacy [21, 22]. Research states that when parents are repeatedly faced with their failed attempts to deal with children’s weight-related problem behaviors, their self-confidence is undermined, and parents are more likely to exacerbate the use of ineffective parenting strategies [21].

The purpose of the current study is to evaluate the psychometric properties of an adapted version of the PAPP questionnaire with parents of school-aged children. Specific objectives include: 1) assessing the factor structure of the PAPP scales in a community sample of parents of school-aged children; 2) testing measurement invariance of the PAPP scales’ factor structures across children’s weight status based on BMI z-scores (thin/normal vs overweight/obese); and 3) evaluating the construct validity by assessing the association between PAPP scales’ factors and related constructs, namely parents’ feeding-related practices and parents’ self-efficacy. Considering the similarities between the PA and feeding practices’ conceptual frameworks, results are expected to reveal significant associations between practices categorized in each domain within the respective conceptual framework [12, 16]. In addition, results are expected to present negative associations between the Discouragement scale’s factors and parents’ self-efficacy in handling children’s lifestyle problem behaviors.

## Method

### Participants

Participants were 503 parents or caregivers of children aged 5 to 10 (*M* = 7.68, *SD* = 1.32) recruited from seven public school clusters in the North of Portugal, one of the regions with the highest prevalence of childhood OW/OB in the country [23]. Most of the participants were mothers (*n* = 428, 85.1%), in average 37.90 years old (*SD* = 5.58), with 11.54 years of schooling (*SD* = 3.90), and with a thin or normal weight status (*n* = 275, 54.7%), as per their report. Most of the children (53.5% female) were enrolled in the second, third or fourth grades (*n* = 302, 60.0%), and had a thin or normal weight status (BMI z-score < = 1 *SD*; *n* = 278, 55.2%), as per their caregivers’ report. Further details on the sociodemographic characteristics of the sample are presented in Table S1 in the Additional file 1.

### Procedure

A cluster sampling strategy was used to obtain random lists of public school clusters that included pre-schools and elementary schools, in each of the six most populated sub-regions of the north region of Portugal, according to the information available in the Database of Contemporary Portugal (PORDATA) [24]. School clusters were then contacted, and seven agreed to take part in the study.

With the collaboration of the schoolteachers, 740 parents/caregivers were invited to participate in the study. Inclusion criteria were being a parent/caregiver of a child aged 5–10 years, and having basic language skills in Portuguese. All participants were informed about the study, and those who agreed to participate signed an informed consent form. Parents completed the printed assessment protocol during school evaluation meetings. Those who could not attend the evaluation meeting received the printed assessment protocol through their child’s classroom teacher, completed the questionnaires at home, and then returned the protocol in a sealed envelope to the research team through the teacher.

A total of 526 parents completed the printed assessment protocol (315 in the schools, in the presence of a researcher, and 211 at home by themselves). Only 503 were considered, as 23 questionnaires were returned blank, or were completed incorrectly. Even though the original study used a 2 weeks period for the test-restest reliability, in the current study, parents were invited to complete the PAPP again 1 month after the first assessment protocol. The longer period for the test-retest reliability evaluation was choosen given that in the measure’s instruction parents are asked to answer about their specific PA parenting practices over the last 30 days. If a shorter period was considered, the possibility of overestimating the temporal stability of the PAPP constructs had to be acknowledged. In addition, the literature on research methods also describes that test-retest reliability can be evaluated in intervals up to 4 weeks [25]. A total of 125 parents completed the questionnaire a second time, on average 32 days (*SD* = 8.72) after the first assessment wave. Data collection took place in 2019, from April to July.

### Measures

#### Sociodemographic characteristics

Parents completed a sociodemographic questionnaire about the sex, age, education, height, and weight of their children, themselves and their partners. Children’s BMI z-scores were estimated according to the WHO reference [26]. Caregiver’s BMI scores were calculated according to the WHO guidelines [27].

#### Physical activity parenting practices (PAPP)

The PAPP was translated into European Portuguese and adapted to be used with parents of school-aged children aged 5–10. Details on the translation and adaptation procedures are described elsewhere [28].

The PAPP is a self-report questionnaire that evaluates the frequency of parental practices that encourage and discourage children’s engagement in PA [7]. The encouragement scale assesses parenting practices encouraging children’s PA and includes a single-factor, Engagement and Structure, with 15 items (e.g., “How often do you go on a walk with your child?”), and two single items (e.g., “How often do you not register your child for sports or dance due to lack of money?). The discouragement scale assesses parenting practices that discourage children’s engagement in PA and includes four factors, Promote Screen Time (3 items; e.g., “(…) allow your child to play a lot of videogames?“), Promote Inactivity (3 items; e.g., “(…) drive your child, when it was easy to walk?“), Psychological Control (5 items; e.g., “(…) discipline your child for being too active?“) and Restriction for Safety Concerns (4 items; e.g., “(…) let your child go outside to play around your home?”). The items are rated on a 5-point Likert scale ranging from 1 (never) to 5 (always) and reflect the parents’ assessment of how often they used each practice in the previous month.

#### Comprehensive feeding practices questionnaire (CFPQ)

The CFPQ is a self-report instrument, that assesses specific parenting practices related to feeding, among parents of children aged between 18 months and 8 years [17]. It was translated into European Portuguese and adapted for use with parents of children aged 5–10 years [29]. The study of its psychometric properties with a Portuguese sample of parents revealed that the questionnaire is a reliable measure to assess feeding-related parenting practices in a nine-factor model structure: Monitoring, Modeling, Promotion of Healthy Eating, Involvement, Child Control, Food as Reward, Emotion Regulation, Pressure to Eat, and Restriction for Weight Control and for Health. Using a 5-point rating scale, participants were asked to indicate how often they use a specific strategy (13 items), from 1 (never) to 5 (always), or the degree to which they agreed with a statement (30 items), from 1(disagree) to 5 (agree). In the present study, the CFPQ [29] revealed acceptable fit to data (χ^2^_807_ = 1363.12; *p* < .001; CFI = .91; TLI = 0.90; RMSEA = 0.04; SRMR = 0.05) and Cronbach’s alpha values ranged from .55 (Food as reward) to .87 (Monitoring).

#### Lifestyle behavior checklist (LBC)

The LBC [30, 31] is a self-report measure with two scales that evaluates parental perceptions of children’s problematic behaviors related to OW and OB (Problem scale) and parental self-efficacy in dealing with those problems (Confidence scale). In the present study only the confidence scale was used. The scale includes 26 statements illustrating problem behaviors related to Overeating (e.g., “Eats too quickly”), Misbehavior in Relation to Food (e.g., “Demands food”), Emotional Correlates of Being Overweight (e.g., “Complains about being teased”) and Physical Activity (e.g., “Watches too much television”), and parents are asked to rate each statement from 1 (certain I can’t do it) to 10 (certain I can do it). In the current study, the Confidence scale revealed acceptable fit to the data (χ^2^_(154)_ = 533.01; *p* < .001; CFI = .90; TLI = .88; RMSEA = .070; SRMR = .073) and Cronbach’s alpha values ranged from .90 (Physical Activity) to .92 (Overeating and Emotional Correlates of Being Overweight).

### Data analysis

Confirmatory factor analyses (CFA) were performed to evaluate the single-factor structure and the four-factor structure of the PAPP’s Encouragement and Discouragement scales, respectively. The dataset included missing data, which appeared to be missing completely at random (MCAR), as described in the results section. To deal with missing data, CFA were performed using the full information maximum likelihood estimation method. For model fit evaluation, CFI > = .95, TLI > = 0.95, RMSEA <= .06, and SRMR ≤ .10 were used as indicators of good fit to the data, CFI values from .90 to .94, TLI values from 0.90 to 0.94 and RMSEA values from .07 to .08 were used as indicators of acceptable fit [32, 33]. Additionally, CFI and TLI values between 0.80 and 0.90, and RMSEA values above 0.08 and equal to or below 0.10, were used as indicators of marginally acceptable fit [32, 33]. To improve model fit, model re-specification allowing residual covariances was performed [34].

Measurement invariance for each factor structure was tested by performing CFA multigroup comparison according to children’s BMI z-score (underweight/normal vs. overweight/obese). Cross-group constraints were set, and the more restricted models compared with the less restricted ones [35], for configural, metric, and scalar invariance. Invariance was considered when ΔCFI ≤ − .01 and ΔRMSEA ≤0.015 [35, 36].

To evaluate the factors’ reliability, Cronbach’s α values were obtained considering .70 or higher as indicative of an adequate reliability [34]. Convergent and divergent validity, and temporal stability were studied using Pearson’s correlation coefficients [37]. For test-retest reliability evaluation, correlation coefficients equal or above .70 were deemed indicative of adequate temporal stability [38].

## Results

### Descriptive statistics

The items’ descriptive statistics are presented in Table S2 in Additional file 2. The items distribution revealed no severe deviations from the normal distribution [34]. The dataset presented a total of 1.51% missing values, 17.7% of incomplete cases, and a range from 0 to 71% of missing data (*M* = 1.51%, *SD* = 5.45). A multiple regression analysis evaluating the sociodemographic and anthropometric characteristics of the children and parents as predictors of the percentage of missing values per participant was performed. Results (adjusted *R*^2^ = 0.02, *F*_(13, 367)_ = 1.73, *p* = .054) showed that none of the variables in the dataset relate to the missing data, suggesting that the missingness pattern is completely at random (MCAR).

### Confirmatory factor analyses (CFA)

**Fig. 1:**
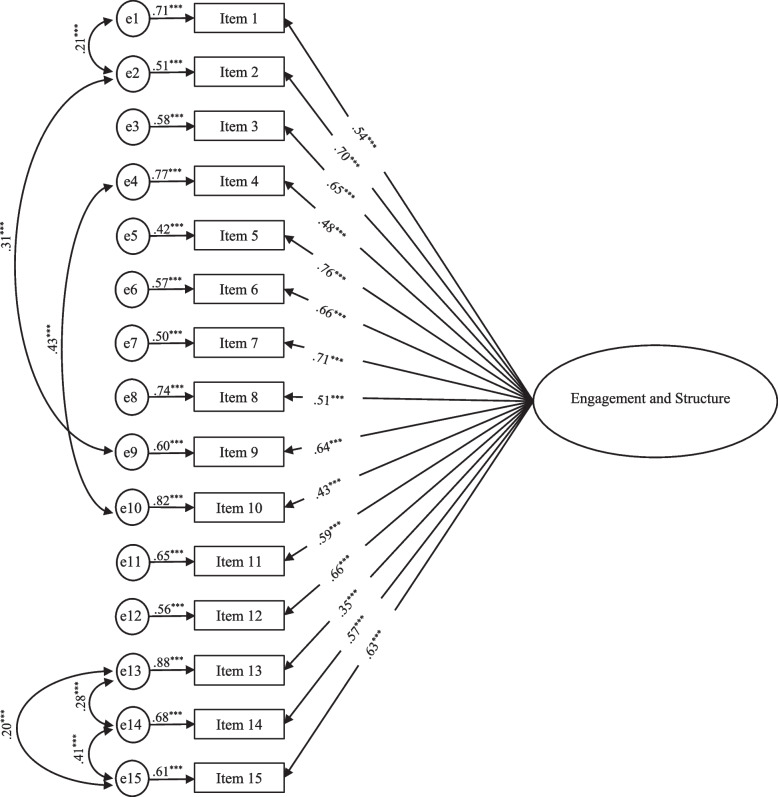
Confirmatory Factor Analysis of the single-factor structure Engagement and Structure, from the PAPP Encouragement scale

The single-factor structure Engagement and Structure (Encouragement scale) was tested with 15 items (Model 1) [7]. The single items of the scale were not included in the model. As presented in Table 1, the model revealed poor fit, *χ*^2^ _(90)_ = 594.08, CFI = .83, TLI = 0.80, RMSEA = .11, SRMR = .06, with standard factor loading values (SFL) ranging from .39 to .74. A second model was then tested (Model 2), including items residual covariance between six pairs of items. Model E2 revealed an acceptable model fit, *χ*^2^_(84)_ = 323.28, CFI = .92, TLI = 0.90, RMSEA = .08, SRMR = .05 (Table 1), with SFL ranging from .35 (item 13) to .76 (item 5) (Fig. 1), and good internal consistency (α = .89).

**Table 1:** Results of the confirmatory factor analysis for the Physical Activity Parenting Practices questionnaire (PAPP)

	χ^2^	*df*	CFI	TLI	RMSEA	SRMR	SFL Range	*α*
Model 1 – PAPP-E	594.08	90.00	.83	0.80	0.11	0.06	.39–.74	.89
Model 2 – PAPP-E	323.28	84.00	.92	0.90	0.08	0.05	.35–.76	.89
Model 3 – PAPP-D	124.27	71.00	.98	0.97	0.04	0.04	−.23 - -.81	.73
Model 4 – PAPP-D	82.85	51.00	.99	0.98	0.04	0.04	−.23 - -.80	.70

*Model 1 – PAPP-E* single-factor structure of the Physical Activity Parenting Practices Encouragement scale, *Model 2 – PAPP-E* single-factor structure of the Physical Activity Parenting Practices Encouragement scale after re-specification, based on the modification indexes, *Model 3 – PAPP-D* the four-factor structure of the Physical Activity Parenting Practices Discouragement scale, *Model 4 – PAPP-D* a three-factor structure version of the Physical Activity Parenting Practices Discouragement scale, after excluding the factor Promote Inactivity; *χ*^2^  Chi-square; *df*  degrees of freedom, *CFI* Comparative Fit Index, *TLI* Tucker- Lewis Index, *RMSEA* Root Mean Square of Approximation, *SRMR* Standardized Root Mean Squared Residual, *SFL* Standard Factor Loading, *α* Cronbach’s alpha

Standardized factor loadings, residual variance and residuals’ correlations for Model 2 – PAPP-E; *p < .05, **p < .01, ***p < .001.

The CFA was then replicated with the data from the second assessment wave (*n* = 125), revealing marginally acceptable fit (*χ*^2^_(84_) = 178.39, CFI = .84, TLI = 0.80, RMSEA = .10, SRMR = .07). The scores of the factor in each assessment wave were computed from the SFL and the correlation coefficient (*r* = .57, *p* < .001) revealed a temporal stability below appropriate for the Encouragement scale.

**Fig. 2:**
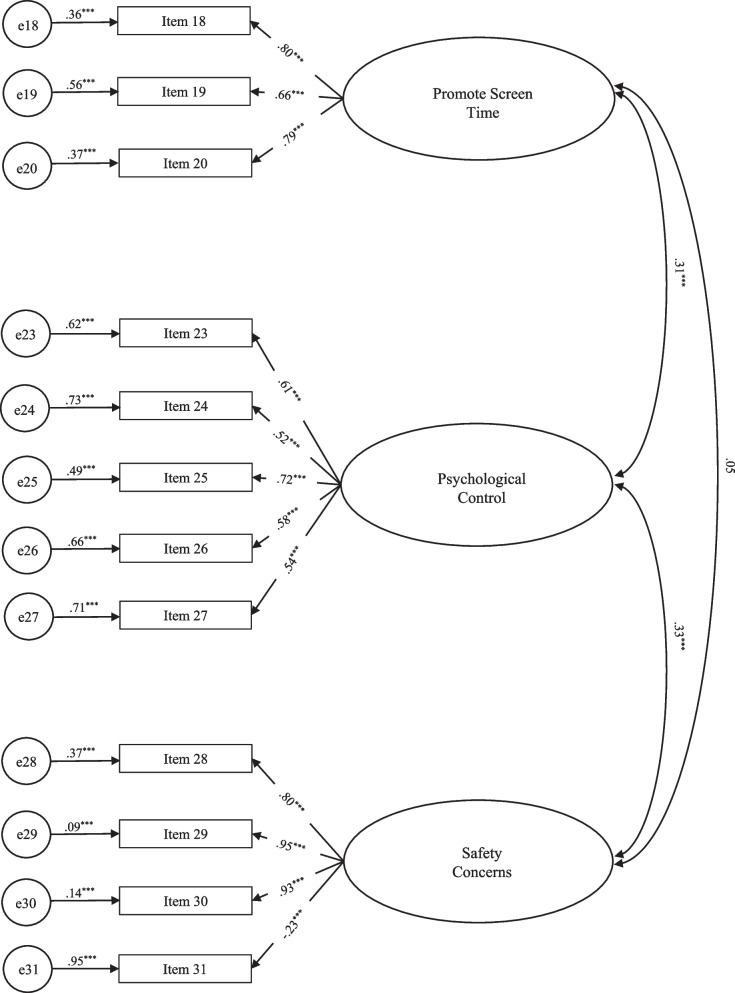
Confirmatory Factor Analysis of the three-factor structure of the PAPP Discouragement scale

The test of the four-factor structure of the discouragement scale included 14 items (Model 3) and revealed good fit to the data, *χ*^2^_(71)_ = 124.27, CFI = .98, TLI = 0.97, RMSEA = .04, SRMR = .04 (Table 1). As the factor Promote Inactivity included only two items, precluding the identification of a factor according to Kline [34], a new CFA was performed excluding this factor from the model (Fig. 2). The final model, with 12 items and 3 factors (Model 4), revealed an excellent fit to the data, *χ*^2^_(51)_ = 82.85, CFI = .99, TLI = 0.98, RMSEA = .04, SRMR = .04 (Table 1). Items' SFL ranged from .52 (item 24) to .95 (item 29), the exception being item 31, which revealed a SFL of −.23, as shown in Fig. 2. Positive moderate correlations were found between Promote Screen Time and Psychological Control (r = .31, *p* < .001), as well as between Psychological Control and Safety Concerns (*r* = .33, *p* < .001). Reliability analysis yielded acceptable internal consistency for Promote Screen Time (α = .79) and Psychological Control (α = .73), and relatively low for Safety Concerns (α = .64). A CFA of the final model was performed with the data from the second assessment wave and revealed good fit to the data (*χ*^2^_(51)_ = 68.26, CFI = .97, TLI = 0.96, RMSEA = .05, SRMR = .06). The test-retest reliability evaluation deemed acceptable temporal stability for the factors Promote Screen Time (*r* = .73, *p* < .001), Psychological Control (*r* = .74, *p* < .001) and Safety Concerns (*r* = .74, *p* < .001).

Standardized factor loadings, residual variance, and latent factors’ correlations for Model 4 – PAPP-D; *p < .05, **p < .01, ***p < .001.

### Measurement invariance

For each of the two final models, measurement invariance was tested by performing CFA multigroup comparisons according to children’s BMI z-score underweight/normal (*n* = 278) vs. overweight/obese (*n* = 189). Configural, metric and scalar invariance were found between groups for encouragement and discouragement scales (Table 2).

**Table 2:** Measurement invariance for the PAPP scales across children’s BMI z-score group

	χ^*2*^*(df)*	CFI	RMSEA	Model comparison	ΔCFI	ΔRMSEA
**PAPP Encouragement scale**						
Configural invariance	388.87 (168)	.915	.075			
Metric invariance	404.302 (183)	.914	.072	1E vs. 2E	−.001	−.003
Scalar invariance	421.318 (198)	.914	.070	2E vs. 3E	−.000	−.002
**PAPP Discouragement scale**						
Configural invariance	146.64 (102)	.978	.043			
Metric invariance	166.72 (114)	.974	.045	1D vs. 2D	−.004	.002
Scalar invariance	188.97 (126)	.968	.046	2D vs. 3D	−.006	.001

*χ*^2^ Chi-square, *df* degrees of freedom, *CFI* Comparative Fit Index, *RMSEA* Root Mean Square of Approximation, *ΔCFI* CFI difference, *ΔRMSEA* RMSEA difference

### Construct validity

The constructs evaluated by the PAPP were tested for convergent and discriminant validity using the LBC and the CFPQ factors, as presented in Table 3. The PAPP’s Engagement and Structure factor revealed small to moderate positive associations with the CFPQ’s Monitoring, Modeling, Promotion of Healthy Eating, Involvement, Pressure to Eat, and Restriction for Weight Control and for Health factors. In addition, the same factor presented a small negative association with the CFPQ’s Child Control.

**Table 3:** Correlations between the PAPP, CFPQ, and LBC factors’ scores

Variable	ES	PST	PC	SC
Monitoring (CFPQ)	.12^*^	−.21^***^	−.20^***^	−.07
Modeling (CFPQ)	.28^***^	−.16^***^	−.04	.02
Promotion of Healthy Eating (CFPQ)	.31^***^	−.25^***^	−.11^*^	.02
Involvement (CFPQ)	.35^***^	−.16^***^	−.01	.08
Child Control (CFPQ)	−.15^***^	.37^***^	.19^***^	.05
Food as Reward (CFPQ)	.08	.24^***^	.32^***^	.16^***^
Emotion Regulation (CFPQ)	.06	.17^***^	.27^***^	.12^**^
Pressure to Eat (CFPQ)	.11^*^	.17^***^	.17^***^	.02
Restriction for Weight Control and for Health (CFPQ)	.09^*^	.0003	.13^**^	.13^**^
Overeating (LBCc)	.05	−.20^***^	−.23^***^	−.10^*^
Misbehavior in Relation to Food (LBCc)	.07	−.22^***^	−.23^***^	−.11^*^
Emotional Correlates of Being Overweight (LBCc)	.05	−.16^***^	−.18^***^	−.08
Physical activity (LBCc)	.05	−.19^***^	−.20^***^	−.09^*^

*PAPP* Physical Activity Parenting Practices, *CFPQ* Comprehensive Feeding Practices Questionnaire, *LBCc* Lifestyle Behavior Checklist confidence scale, *ES* Engagement and Structure (PAPP Encouragement scale), *PST* Promote Screen Time (PAPP Discouragement scale), *PC* Psychological Control (PAPP Discouragement scale), *SC* Safety Concerns (PAPP Discouragement scale); *p < .05, **p < .01, *** *p* < .001

The Promote Screen Time factor revealed small negative associations with CFPQ’s Monitoring, Modeling, Promotion of Healthy Eating, Involvement, as well as with the LBC confidence scale factors of Overeating, Misbehavior in Relation to Food, Emotional Correlates of Being Overweight, and Physical Activity. The same factor revealed small to moderate positive associations with the CFPQ’s Child Control, Food as Reward, Emotion Regulation, and Pressure to Eat.

The Psychological Control factor revealed small negative associations with CFPQ’s Monitoring, Modeling, Promotion of Healthy Eating, and also with the LBC confidence scale factors. The same factor revealed small to moderate positive associations with the CFPQ’s Child Control, Food as Reward, Emotion Regulation, Restriction for Weight Control and for Health factors and Pressure to Eat.

Finally, the Safety Concerns revealed small positive associations with the CFPQ’s Food as Reward, Emotion Regulation, and Restriction for Weight Control and for Health factors, and negative associations with the LBC confidence scale factors of Overeating, Misbehavior in Relation to Food, and Physical Activity.

## Discussion

The current study evaluated the psychometric properties of the Portuguese version of the PAPP questionnaire in a sample of school-aged children. The study addressed the factor structure of the encouragement and discouragement scales, evaluated the scales’ factor-structure measurement invariance across two groups of children according to their weight status, and assessed its constructs’ validity.

The results for the PAPP’s single-factor Encouragement and Structure, which integrates the Encouragement scale, revealed a factor structure similar to the one described by the original authors [7]. Nevertheless, test-retest reliability in the current study was lower. This difference can stem from the larger time interval between assessments in the current study compared to previous research (about 14 days) [7].

An excellent fit was found for the three-factor Discouragement scale (Promote Screen Time, Psychological Control, and Safety Concerns). In the adaptation of the questionnaire to parents of school-age children, the item related to the use of a stroller was discarded, and as a consequence the factor Promoting Inactivity was not included in the model because with only two items, the factor could not be estimated [34].

The three-factor model structure kept the original items composition and presented acceptable reliability, with the exception of the factor Restriction for Safety Concerns, which revealed the lowest internal consistency. This may be consequence of the low SFL of item 31 (“How often do you let your child go outside to play around your home?”) which in turn, may be related with some heterogeneity of participants’ answers according to their area of residence and type of housing (e.g., house with/without a garden). These characteristics can potentially discourage PA parenting practices, especially for those living in urban areas, therefore future studies should explore how the area of residence and type of housing impact the child’s autonomous activity/play around their homes.

The current study’s results also found configural, metric, and scalar invariance across parents of children of different weight statuses (underweight/normal vs. overweight/obese) for the factor structure of both PAPP scales. Such findings indicate that the parents assessed the constructs in the scales similarly, regardless of the weight status of their child. With these results, it can be ascertained that the PAPP, as an instrument, can distinguish the PA parenting practices of parents of children with different weight statuses. These findings show the utility of the questionnaire for research and intervention purposes.

The study’s results also suggest the validity of the constructs assessed by the PAPP’s scales. Parents’ PA and feeding practices were found to be related, which is consistent with the research showing the importance of a balance between energy consumption and expenditure when adopting a healthy lifestyle [3]. As expected, due to the similarities between the PA [12] and feeding practices [16] frameworks, small associations between PA and feeding parenting practices were found, suggesting that these take place within a pattern of care which is part of the families’ lifestyles. Parents who used more practices encouraging PA also reported a higher use of adequate feeding practices, such as Monitoring, Modeling, Promotion of Healthy Eating, Involvement, and Restriction for Weight Control and for Health, placed in the structure and in the autonomy support domains of Vaughn et al. content map [16]. Also, parents who used more often PA practices encouraging children’s engagement in PA used less inadequate feeding practices, such as Child Control but more Pressure to Eat (lower bound of the structure domain [16], reflecting higher levels of permissiveness in children’s feeding). The association between the practices encouraging PA and Pressure to Eat, albeit small, was unexpected. However, as the construct Pressure to Eat addresses the quantity, and not the quality, of the food eaten by the child [17], it is not possible to determine to which type of food(s) the parents were referring to when they answered the CFPQ’s items.

Concerning parenting practices discouraging children’s engagement in PA, the findings suggest that the parents who use more often practices placed in the Neglect/Control domain of PA practices [12], such as Promote Screen Time and Psychological Control, also tend to use less adequate and more inadequate feeding practices. Specifically, parents who revealed higher levels of Promote Screen Time report less Monitoring, Modeling, Promotion of Healthy Eating, and Involvement, and use more practices of Child Control in relation to feeding, Food as Reward, Emotional Regulation through food, and Pressure to Eat. A similar pattern of associations was found regarding Psychological Control, with the parents who report higher levels of this variable, also reporting less Monitoring and Promotion of Healthy Eating, and more inadequate practices of Child Control in relation to feeding, Food as Reward, Emotional Regulation through food, Pressure to Eat, and Restriction. Finally, parents who reported more Safety Concerns also reported more inadequate feeding practices of Food as Reward, Emotional Regulation through food and Restriction.

The associations between discouraging PA and inadequate feeding practices, framed within the Neglect/Control domain of PA practices [12] and the Structure domain of feeding practices [16] may reflect low parental control and unstructured parenting practices related with parental permissiveness, or even lack of parental supervision and guidance, which can contribute to the maintenance of a poor lifestyle, and as a consequence, of children’s overweight/obesity. The associations between specific PA and feeding parenting practices should be a focus of attention in future research, specifically evaluating how parents combine them and how such combinations affect children’s (un)healthy behaviors. Such findings would be relevant contributions to the literature on the profiles of school-aged children’s lifestyle behaviors and their association with childhood overweight and obesity [39, 40].

In the current study, discouraging PA practices were negatively related to parents’ confidence to deal with child’s lifestyle behavior problems. This is consistent with previous research suggesting that ineffective parenting strategies are linked to lower levels of parental self-confidence; e.g., parents who use psychological control practices related to PA and promote more screen time feel less confident when facing difficult lifestyle situations [21].

Parents, as main caregivers, are responsible for most decisions related to children’s lifestyle. Practices regarding children’s PA and feeding are related, reflected in the pattern of care provided by the parents and, to some extent, characterize families’ lifestyles. The findings suggest that by promoting encouraging and preventing discouraging PA parenting practices, parents may adopt healthier, and reduce inadequate feeding practices, increasing their self-efficacy in dealing with children’s lifestyle behavior problems. Nevertheless, given the cross-sectional and correlational nature of the study, conclusions about the directionality of the associations cannot be drawn and should be focus of attention in future prospective research.

The current study was the first to use a version of the PAPP adapted to evaluate PA parenting practices with parents or caregivers of school-aged children. Other strengths of the current study include the sample size, the rigorous procedures undertaken to ensure a distributed sample, and the analytic plan which included structural equation modelling. However, there are also some limitations. A first one relates to mothers being the main informants. Given the well-known influence of fathers over their children’s PA, the inclusion of more fathers as informants would be desirable to study measurement invariance across parents. A second limitation regards the use of self-report measures only. Future studies should collect data using other data collection methods (e.g., interviews), including multiple informants, and also assessing the characteristics of the neighborhood that can influence PA parenting practices (e.g., housing type, traffic, playgrounds, and other community facilities).

## Conclusion

The Portuguese version of PAPP comprises a single-factor Encouragement scale and a three-factor Discouragement scale. Overall, the instrument revealed good psychometric properties in a Portuguese sample and seemed to be a reliable and valid measure to assess PA parenting practices among parents of children aged 5–10. The constructs assessed had the same meaning for parents, regardless of their children’s weight status. The PAPP construct validity was supported by its associations with parents’ feeding practices and parents’ confidence to address children’s lifestyle behaviors. The Portuguese version of the PAPP can thus be useful for both research and practice focusing on childhood OW/OB.

### Supplementary Information

**Additional file 1 : Table S1.** Sociodemographic characteristics of the sample (*N* = 503).

**Additional file 2 : Table S2.** Descriptive statistics for each PAPP item (*N*=503).

**Additional file 3.** STROBE Statement checklist of items that should be included in reports of cross-sectional studies.

**Additional file 4.** Sampling strategy, recruitment process, and strategy to handle missing data.

## Data Availability

The datasets analyzed during the current study are available in the Open Science Framework repository, osf.io/qctuh.

## Abbreviations

OW: Overweight
OB: Obesity
OW/OB: Overweight and obesity
PA: Physical activity
BMI: Body mass index
PAPP: Physical Activity Parenting Practices
PAPP-E: Physical Activity Parenting Practices Encouragement Scale
PAPP-D: Physical Activity Parenting Practices Discouragement Scale
M: Mean
SD: Standard deviation
PORDATA: Database of Contemporary Portugal
WHO: World Health Organization
CFPQ: Comprehensive Feeding Practices Questionnaire
CFI: Comparative Fit Index
TLI: Tucker-Lewis Index
RMSEA: Root Mean Square Error of Approximation
SRMR: Standardized Root Mean Square Residual
LBC: Lifestyle Behavior Checklist
CFA: Confirmatory factor analyses
MCAR: Missing Completely at Random
SFL: Standard Factor Loading Values
LBCc: Lifestyle Behavior Checklist Confidence Scale
ES: Engagement and Structure
PST: Promote Screen Time
PC: Psychological Control
SC: Safety Concerns
