# Supplementary material for: Psychometric properties of the Portuguese version of the physical activity parenting practices questionnaire
Source: BMC Psychol. 2023 Nov 28;11:417. doi: 10.1186/s40359-023-01444-4 (PMC10683127; doi:10.1186/s40359-023-01444-4)
Supplement: Supplementary file 1 — Additional file 1 : Table S1. Sociodemographic characteristics of the sample (N = 503). [file 40359_2023_1444_MOESM1_ESM.docx]

**Additional file 1**

**Table S1** Sociodemographic characteristics of the sample (*N* = 503).

| Informant | *M* | | *SD* |
| --- | --- | --- | --- |
| Age | 37.90 | | 5.58 |
| Education (years) | 11.54 | | 3.90 |
| Relationship with the child | *n* | | *%* |
| Mother | 428 | | 85.1 |
| Father | 62 | | 12.3 |
| Other | 8 | | 1.6 |
| *Missing* | 5 | | 1.0 |
| BMI |  | |  |
| Thin/Normal | 275 | | 54.7 |
| Overweight/Obese | 209 | | 41.6 |
| *Missing* | 19 | | 3.2 |
| Informant’s partner | *M* | | *SD* |
| Age | 40.06 | | 5.52 |
| Education (years) | 10.20 | | 3.86 |
| BMI | *n* | | *%* |
| Thin/Normal | 190 | | 37.8 |
| Overweight/Obese | 242 | | 48.1 |
| *Missing* | 71 | | 14.1 |
| Children | *M* | | *SD* |
| Age | 7.68 | | 1.35 |
| Sex | *n* | | *%* |
| Male | 234 | | 46.5 |
| Female | 269 | | 53.5 |
| BMI z-score |  | |  |
| Thin /Normal | 278 | | 55.2 |
| Overweight /Obese | 189 | | 37.6 |
| *Missing* | 36 | | 7.2 |
| Education |  | | |
| Preschool | 55 | 10.9 | |
| 1^st^ year | 85 | 16.9 | |
| 2^nd^ year | 145 | 28.8 | |
| 3^rd^ year | 112 | 22.3 | |
| 4^th^ year | 105 | 20.9 | |
| *Missing* | 1 | 0.2 | |
| Means (M), Standard deviations (SD), Frequencies (n), and Percentages (%). | | | |
