# Supplementary material for: Psychometric properties of the Portuguese version of the physical activity parenting practices questionnaire
Source: BMC Psychol. 2023 Nov 28;11:417. doi: 10.1186/s40359-023-01444-4 (PMC10683127; doi:10.1186/s40359-023-01444-4)
Supplement: Supplementary file 2 — Additional file 2 : Table S2. Descriptive statistics for each PAPP item (N=503). [file 40359_2023_1444_MOESM2_ESM.docx]

**Additional file 2**

**Table S2** Descriptive statistics for each PAPP item (*N*=503).

| Item | *M* | *SD* | Range | Skewness | Kurtosis |
| --- | --- | --- | --- | --- | --- |
| 1 | 3.16 | 1.10 | 1 - 5 | 0.01 | -0.54 |
| 2 | 3.36 | 0.91 | 1 - 5 | 0.09 | 0.03 |
| 3 | 3.52 | 0.85 | 1 - 5 | 0.20 | -0.34 |
| 4 | 4.29 | 0.79 | 2 - 5 | -0.90 | 0.17 |
| 5 | 3.27 | 0.88 | 1 - 5 | 0.17 | -0.26 |
| 6 | 3.56 | 0.91 | 1 - 5 | -0.02 | -0.55 |
| 7 | 3.57 | 0.93 | 1 - 5 | -0.02 | -0.62 |
| 8 | 3.02 | 1.03 | 1 - 5 | 0.09 | -0.32 |
| 9 | 2.85 | 1.04 | 1 - 5 | 0.19 | -0.36 |
| 10 | 4.34 | 0.78 | 1 - 5 | -1.09 | 1.14 |
| 11 | 3.62 | 0.78 | 1 - 5 | 0.05 | -0.23 |
| 12 | 3.62 | 0.84 | 2 - 5 | 0.03 | -0.65 |
| 13 | 3.90 | 1.21 | 1 - 5 | -0.82 | -0.40 |
| 14 | 3.52 | 0.93 | 1 - 5 | -0.13 | -0.31 |
| 15 | 3.23 | 0.98 | 1 - 5 | 0.09 | -0.30 |
| 16 | 1.88 | 1.17 | 1 - 5 | 1.12 | 0.20 |
| 17 | 3.84 | 1.14 | 1 - 5 | -0.66 | -0.47 |
| 18 | 2.70 | 0.78 | 1 - 5 | 0.54 | 0.85 |
| 19 | 2.34 | 0.84 | 1 - 5 | 0.35 | 0.23 |
| 20 | 2.63 | 0.78 | 1 - 5 | 0.39 | 0.67 |
| 21 | 1.61 | 0.84 | 1 - 5 | 1.45 | 2.07 |
| 22 | 2.25 | 0.97 | 1 - 5 | 0.47 | -0.08 |
| 23 | 1.44 | 0.72 | 1 - 5 | 1.93 | 4.36 |
| 24 | 1.30 | 0.76 | 1 - 5 | 2.87 | 8.10 |
| 25 | 1.64 | 0.89 | 1 - 5 | 1.34 | 1.43 |
| 26 | 1.66 | 0.86 | 1 - 5 | 1.10 | 0.43 |
| 27 | 1.61 | 0.89 | 1 - 5 | 1.39 | 1.34 |
| 28 | 2.58 | 1.36 | 1 - 5 | 0.44 | -0.94 |
| 29 | 2.77 | 1.45 | 1 - 5 | 0.27 | -1.22 |
| 30 | 2.92 | 1.43 | 1 - 5 | 0.14 | -1.24 |
| 31 | 3.22 | 1.36 | 1 - 5 | -0.22 | -1.11 |

Mean (M), Standard deviation (SD)
