# Supplementary material for: Psychometric properties of the Portuguese version of the physical activity parenting practices questionnaire
Source: BMC Psychol. 2023 Nov 28;11:417. doi: 10.1186/s40359-023-01444-4 (PMC10683127; doi:10.1186/s40359-023-01444-4)
Supplement: Supplementary file 3 — Additional file 3. STROBE Statement checklist of items that should be included in reports of cross-sectional studies. [file 40359_2023_1444_MOESM3_ESM.docx]

**Additional file 3**

STROBE Statement checklist of items that should be included in reports of cross-sectional studies.

|  | Item No | Recommendation | Check |
| --- | --- | --- | --- |
|  |  |  |  |
| **Title and abstract** | 1 | (*a*) Indicate the study’s design with a commonly used term in the title or the abstract | *The type of study is indicated in the title.* |
|  |  | (*b*) Provide in the abstract an informative and balanced summary of what was done and what was found | *The abstract gives a summary of the study.* |
| Introduction | | |  |
| Background/rationale | 2 | Explain the scientific background and rationale for the investigation being reported | *Background and rationale are*  *Reported.* |
| Objectives | 3 | State specific objectives, including any prespecified hypotheses | *Aims are detailed in the Introduction.* |
| Methods | | |  |
| Study design | 4 | Present key elements of study design early in the paper | *Key elements are reported.* |
| Setting | 5 | Describe the setting, locations, and relevant dates, including periods of recruitment, exposure, follow-up, and data collection | *Setting is described.* |
| Participants | 6 | (*a*) Give the eligibility criteria, and the sources and methods of selection of participants | *Eligibility criteria and methods of selection are detailed.* |
| Variables | 7 | Clearly define all outcomes, exposures, predictors, potential confounders, and effect modifiers. Give diagnostic criteria, if applicable | *All the outcome variables*  *are described. Diagnostic criteria for children’s BMI z-scores were based on the WHO growth charts.* |
| Data sources/ measurement | 8* | For each variable of interest, give sources of data and details of methods of assessment (measurement). Describe comparability of assessment methods if there is more than one group | *Psychometric characteristics of questionnaires are reported. Criteria for comparability of groups are reported.* |
| Bias | 9 | Describe any efforts to address potential sources of bias | *Reporting bias deriving from incomplete answer was addressed by studying missingness and by using appropriate methods to deal with the missing pattern found.*  *To reduce the sampling bias associated with the use of a convenience sample, and to increase the sample representativeness we used a two-stage cluster sampling strategy.* |
| Study size | 10 | Explain how the study size was arrived at | *The data were collected in the context of a broader study, that requires a higher sample size. For the current study we ensured a minimum ratio of 10 observations per observed variable, based on the rule of thumb of Nunnally (1967).* |
| Quantitative variables | 11 | Explain how quantitative variables were handled in the analyses. If applicable, describe which groupings were chosen and why | *Method of handling variables was reported. The criteria for selecting groups were detailed.* |
| Statistical methods | 12 | (*a*) Describe all statistical methods, including those used to control for confounding | *Statistical methods were described.* |
|  |  | (*b*) Describe any methods used to examine subgroups and interactions | *Statistical methods were described.* |
|  |  | (*c*) Explain how missing data were addressed | *The dataset included missing data, which appeared to be MCAR. To deal with this, we used structural equation modelling with the full information maximum likelihood estimation method.* |
|  |  | (*d*) If applicable, describe analytical methods taking account of sampling strategy | *Statistical methods were described.* |
|  |  | (*e*) Describe any sensitivity analyses | *Not applicable.* |
| Results | | |  |
| Participants | 13* | (a) Report numbers of individuals at each stage of study—eg numbers potentially eligible, examined for eligibility, confirmed eligible, included in the study, completing follow-up, and analysed | *Number of participants is reported.* |
|  |  | (b) Give reasons for non-participation at each stage | *Participation was voluntary.*  *Reasons for non-participation were described in detail in Additional file 4.* |
|  |  | (c) Consider use of a flow diagram | *Not applicable.* |
| Descriptive data | 14* | (a) Give characteristics of study participants (eg demographic, clinical, social) and information on exposures and potential confounders | *Characteristics are reported and analysed.* |
|  |  | (b) Indicate number of participants with missing data for each variable of interest | *Missing date for the variables of interest were described.* |
| Outcome data | 15* | Report numbers of outcome events or summary measures | *Numbers are reported.* |
| Main results | 16 | (*a*) Give unadjusted estimates and, if applicable, confounder-adjusted estimates and their precision (eg, 95% confidence interval). Make clear which confounders were adjusted for and why they were included | *Not applicable.* |
|  |  | (*b*) Report category boundaries when continuous variables were categorized | *Children’s BMI z-score was categorized.* |
|  |  | (*c*) If relevant, consider translating estimates of relative risk into absolute risk for a meaningful time period. | *Not applicable* |
| Other analyses | 17 | Report other analyses done—eg analyses of subgroups and interactions, and sensitivity analyses | *All analyses done were reported.* |
| Discussion | | |  |
| Key results | 18 | Summarise key results with reference to study objectives | *Key results are summarized.* |
| Limitations | 19 | Discuss limitations of the study, taking into account sources of potential bias or imprecision. Discuss both direction and magnitude of any potential bias | *Limitations of the study are discussed.* |
| Interpretation | 20 | Give a cautious overall interpretation of results considering objectives, limitations, multiplicity of analyses, results from similar studies, and other relevant evidence | *The interpretation of the results was very cautious, given the cross-sectional nature of the study which does not allow to infer causality.* |
| Generalisability | 21 | Discuss the generalisability (external validity) of the study results | *The generalisability was discussed.* |
| Other information | | |  |
| Funding | 22 | Give the source of funding and the role of the funders for the present study and, if applicable, for the original study on which the present article is based | *The information is provided.* |

*Give information separately for exposed and unexposed groups.

**Note:** An Explanation and Elaboration article discusses each checklist item and gives methodological background and published examples of transparent reporting. The STROBE checklist is best used in conjunction with this article (freely available on the Web sites of PLoS Medicine at http://www.plosmedicine.org/, Annals of Internal Medicine at http://www.annals.org/, and Epidemiology at http://www.epidem.com/). Information on the STROBE Initiative is available at www.strobe-statement.org.
