# Supplementary material for: Psychometric properties of the Portuguese version of the physical activity parenting practices questionnaire
Source: BMC Psychol. 2023 Nov 28;11:417. doi: 10.1186/s40359-023-01444-4 (PMC10683127; doi:10.1186/s40359-023-01444-4)
Supplement: Supplementary file 4 — Additional file 4. Sampling strategy, recruitment process, and strategy to handle missing data. [file 40359_2023_1444_MOESM4_ESM.docx]

**Additional file 4**

Sampling strategy, recruitment process, and strategy to handle missing data.

**Sampling and recruitment**

To collect data from a community sample of parents of 5-to-10 year-olds in the north of Portugal, we choose to contact the public schools of the Northern region of Portugal. To obtain a geographically balanced sample, we used the latest information available in the Database of Contemporary Portugal (PORDATA) on the distribution of the children attending preschool or elementary school in the Portuguese public school system. We used the frequencies found for the eight sub-regions of the north of Portugal as orientation guides for the participants' selection: Alto Minho (6.5 %), Cávado (11.9 %), Ave (11.2%), Porto (47.5 %), Alto Tâmega (1.8 %), Tâmega e Sousa (13.9 %), Douro (4.9 %) and Terras de Trás-os-Montes (2.4 %). Given that the target population in Alto Tâmega and Terras de Trás-os-Montes was less than 4.5 %, we did not include those regions in the data collection process. We then used a cluster sampling strategy in each of the six sub-regions to obtain six random lists of school clusters, one per sub-region. Afterward, we contacted each school cluster about the study. All schools accepted the invitation to take part. The contacts established with the schools followed the order of the random lists obtained for each sub-region until the percentage of children in each sub-region was approximately achieved in the total sample.

A total of 740 parents were invited to participate. After the informed consent was obtained, participants completed the assessment protocol. Data were collected in two ways: during the evaluation meetings at school, in the presence of a research team member, or at home. The parents who could not attend the evaluation meeting received the assessment protocol through their child's classroom teacher, completed the questionnaires at home, and then returned the protocol in a sealed envelope to the research team through the teacher. The research team provided contacts for the parents completing the assessment protocol at home to reach out and clarify any questions about the study or the assessment protocol.

A total of 526 parents completed a printed version of the assessment protocol (315 in the schools, in the presence of a researcher, and 211 at home by themselves). Only 503 were considered, as 23 questionnaires were returned blank, or were completed incorrectly. Parents were invited to complete the PAPP again one month after recruitment. A total of 125 parents completed the questionnaire a second time, on average 32 days (*SD* = 8.72) after the first assessment wave. Data collection took place in 2019, from April to July.

**Missing data**

The dataset had 1.51% missing values, 17.7% of incomplete cases, and a range from 0% to 71% of missing data (*M* = 1.51%, *SD* = 5.45). A multiple regression analysis was performed, including the children's and parents' sociodemographic and anthropometric characteristics as predictors of the percentage of missing values per participant (adjusted *R*^2^ = 0.02, *F*_(13, 367)_ = 1.73, *p* = .054). Results showed that none of the variables in the dataset relate to the missing data, suggesting that the missingness pattern is completely at random (MCAR). We used structural equation modeling with full information maximum likelihood estimation method to deal with this.
